# The impact of down-regulated *SK3* expressions on Hirschsprung disease

**DOI:** 10.1186/s12881-018-0539-3

**Published:** 2018-02-13

**Authors:** Gunadi, Mukhamad Sunardi, Nova Yuli Prasetyo Budi, Alvin Santoso Kalim, Kristy Iskandar, Andi Dwihantoro

**Affiliations:** 1 Pediatric Surgery Division, Department of Surgery, Faculty of Medicine, Universitas Gadjah Mada/Dr. Sardjito Hospital, Yogyakarta, 55281 Indonesia; 2 Department of Child Health, Faculty of Medicine, Universitas Gadjah Mada/UGM Academic Hospital, Yogyakarta, 55291 Indonesia

**Keywords:** Appropriate pull-through, Hirschsprung disease, Indonesia, Persistent bowel symptoms, *SK3*

## Abstract

**Background:**

Some Hirschsprung’s disease (HSCR) patients showed persistent bowel symptoms following an appropriately performed pull-through procedure. The mechanism is presumed to be down-regulated *small-conductance calcium-activated potassium channel 3 (SK3)* expression in the HSCR ganglionic intestines. We aimed to investigate the *SK3* expression’s impact in HSCR patients after a properly performed pull-through surgery in an Indonesian population, a genetically distinct group within Asia.

**Methods:**

We assessed *SK3* gene expression in both the ganglionic and aganglionic colon of HSCR patients and controls colon by quantitative real-time polymerase chain reaction (RT-PCR).

**Results:**

We ascertained fourteen sporadic HSCR patients and six anorectal malformation patients as controls. Quantitative RT-PCR showed that the *SK3* expression was significantly lower (23-fold) in the ganglionic colon group compared to the control group (9.9 ± 4.6 vs. 5.4 ± 3.4; *p* = 0.044). The expression of *SK3* in the aganglionic colon group was also significantly lower (43-fold) compared to the control group (10.8 ± 4.4 vs. 5.4 ± 3.4; *p* = 0.015).

**Conclusion:**

Our study shows that the down-regulated *SK3* expression in ganglionic intestines might contribute to the persistent bowel symptoms following a properly performed pull-through surgery in Indonesian HSCR patients. Furthermore, this study is the first report of *SK3* expression in a sample population of Asian ancestry.

## Background

Hirschsprung disease (HSCR: MIM# 142623) is a neurodevelopmental disorder characterized by the absence of ganglion cells, resulting in a functional intestinal obstruction in infants. According to the type of aganglionosis, HSCR is classified as follows: short-segment HSCR, long-segment HSCR, and total colon aganglionosis [1, 2]. Its incidence differs among race with 15, 21, and 28 cases per 100,000 live births in European, African and Asian ancestry cases, respectively [1, 2]. It might relate to the *RET* rs2435357 susceptibility allele frequency difference across populations [3, 4]. Our previous studies supported this hypothesis since the frequency of rs2435357 variant in Indonesian ancestry cases is higher than those of European ancestry cases (0.50 vs. 0.25) [5, 6].

The current treatment for Hirschsprung disease (HSCR) is surgical resection of the aganglionic segment of the intestines. Most HSCR patients have a satisfactory outcome after a properly performed pull-through operation, however some patients continue to have persistent bowel symptoms such as constipation, soiling and enterocolitis. The cause of persistent bowel symptoms might be the transition zone pull-through or residual aganglionosis, but many have no identifiable cause for their ongoing bowel dysfunction [7, 8]. The prevailing hypothesis is the down-regulated *small-conductance calcium-activated potassium channel 3 (SK3)* expression in ganglionic bowel in HSCR patients [9]. The *SK3* channels have been shown to be involved in the outward currents activated by purines in the intact muscles in response to enteric inhibitory neurotransmission [10]. It is highly expressed in the platelet-derived growth factor receptor alpha-positive (PDGFRA+) cells [11], which together with interstitial cells of Cajal and smooth muscle cells regulate intestinal secretory activities and peristalsis [10]. In addition, a different genetic characteristic was previously revealed between the Indonesian and European populations with HSCR [12]. Therefore, we aimed to investigate the *SK3* expression in HSCR patients after a properly performed definitive surgery in an Indonesian population, a genetically distinct group within Asia.

## Methods

### Subjects

This cohort study was performed between August 2015 and July 2016 at Dr. Sardjito Hospital, Yogyakarta, Indonesia. The inclusion criteria were children with the age of < 18 years old who have a diagnosis of HSCR based on clinical findings, contrast enema and histopathology, while the exclusion criteria were HSCR patients with low quality of total RNA. We used hematoxylin and eosin staining and S100 immunohistochemistry for histopathology assessment [5, 6, 12–14].

The HSCR patients and controls were ascertained for this study after their parents signed a written informed consent form. The ganglionic and aganglionic intestinal specimens were collected at pull-through operation from HSCR patients, while control intestinal specimens were obtained at colostomy closure from anorectal malformation (ARM) patients. The ganglionic colon specimens were collected at least 10 cm above the transition zone. Intraoperative pathological evaluation was performed during pull-through procedure to ensure that the sample for RNA extraction is from aganglionic (or ganglionic) colon. The ARM patients were chosen as controls according to previous study [9].

The study was reviewed and approved by the Institutional Review Board of the Faculty of Medicine, Universitas Gadjah Mada/Dr. Sardjito Hospital, Indonesia (KE/FK/713/EC/2015).

### RET rs2435357 variant genotyping

Genomic DNA samples of HSCR patients were genotyped for the *RET* rs2435357 variant using the PCR-RFLP method as described in our previous study [5].

### RNA extraction and quantitative RT-PCR

Total RNA was isolated from 25 to 30 mg of colon tissue using the total RNA Mini Kit (Tissue) (Geneaid Biotech Ltd., New Taipei City, Taiwan). The RNA was quantified by a NanoDrop 2000 Spectrophotometer (Thermo Scientific, Wilmington, DE, USA) and immediately stored at − 80°C. The OD260/280 ratios typically range from 1.8 to 2.0, indicating high RNA purity.

The *SK3* expression was quantified using 100 ng of total RNA, the Kapa SBYR Fast qRT-PCR One Step Kit Universal (Kapa Biosystems, Massachusetts, USA), and the BioRad CFX Real-Time PCR System (California, USA). The *SK3* primers were 5′- TGGACACTCAGCTCACCAAG-3′ (forward) and 5’-GTTCCATCTTGACGCTCCTC-3′ (reverse) [15]. *Glyceraldehyde-3-phosphate dehydrogenase (GAPDH)*, a housekeeping gene, was used as an endogenous control. The *GAPDH* primers were 5’-GCACCGTCAAGGCTGAGAAC-3′ (forward) and 5’-TGGTGAAGACGCCAGTGGA-3′ (reverse). The Livak (2^-ΔΔC^_T_) method was utilized to determine the *SK3* mRNA expression level in ganglionic and aganglionic colon from HSCR patients normalizing to *GAPDH* and relative to ganglionic colon from control individuals [16].

### Statistical analysis

Results were expressed as mean values ± SD. The analysis of t-test was used to search for statistical differences between the two groups. A *p* value less than 0.05 was considered statistically significant.

## Results

During a 1-year period of study, we recruited 16 HSCR patients and eight controls according to the inclusion criteria. We excluded two HSCR patients and two controls because of their low quality of total RNA, thus, we further analyzed 14 HSCR patients and six controls. Neither familial nor syndromic HSCR patients were involved in this study.

According to degree of aganglionosis, 86% of patients have a HSCR short-segment. The mean age at diagnosis and definitive surgery was 10.1 ± 31.6 months and 20.4 ± 35.9 months, respectively (Table 1). As for definitive surgery, 57% HSCR patients underwent the transanal endorectal pull-through, followed by the Duhamel and Soave pull-through procedures in 29% and 14% children, respectively (Table 1).

**Table 1:** Clinical characteristics of Indonesian HSCR patients following definitive surgery and controls

Patient	Age at Diagnosis (months)	Type of Aganglionosis	Colostomy	Age at Definitive Surgery (months)	Definitive Surgery	Persisten Bowel Symptoms	*RET* rs2435357 Genotyping
1	0–12	Long	Yes	13–24	Duhamel	–	TT
2	0–12	Short	–	0–12	TEPT	–	CC
3	0–12	Short	–	0–12	TEPT	–	TT
4	0–12	Short	–	0–12	TEPT	Yes	TT
5	0–12	Short	Yes	13–24	Soave	–	TT
6	0–12	Short	Yes	49–60	Duhamel	Yes	TT
7	109–120	Short	Yes	121–132	Duhamel	–	TT
8 9 10 11 12 13 14	0–12 0–12 0–12 0–12 0–12 0–12 0–12	Long Short Short Short Short Short Short	Yes - Yes - - - -	13–24 0–12 13–24 0–12 0–12 0–12 0–12	Soave TEPT Duhamel TEPT TEPT TEPT TEPT	- - - Yes - - -	TT TT TT TT TT TT CC
Control	Age at Stoma Closure (months)	Diagnosis					
1	133–144	ARM					
2	25–36	ARM					
3	13–24	ARM					
4	37–48	ARM					
5 6	109–120 13–24	ARM ARM					

*ARM*, anorectal malformation; *TEPT*, transanal endorectal pull-through

First, we genotyped HSCR patients for the *RET* rs2435357 variant since this variant has been strongly associated with HSCR in an Indonesian population [5, 6]. The genotype frequencies for *RET* rs2435357 variant among HSCR patients were as follows: TT (12/14, 86%), CT (0), and CC (2/14, 14%).

**Fig. 1:**
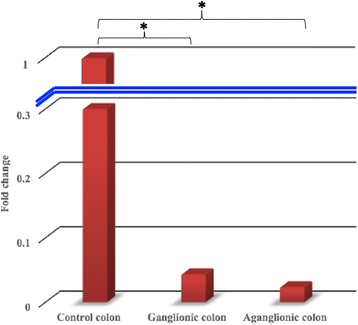
The *SK3* expressions in both the ganglionic and aganglionic colon of HSCR patients are significantly lower (23 and 43-fold, respectively) compared with the controls colon. Data represent the means ± SD of *SK3* expression normalized to *GAPDH* and relative to control tissues. *, *p* < 0.05

Quantitative RT-PCR showed that the *SK3* expression was significantly lower (23-fold) in the ganglionic colon group compared to the control group (9.9 ± 4.6 vs. 5.4 ± 3.4; *p* = 0.044) (Fig. 1). The expression of *SK3* in the aganglionic colon group was also significantly lower (43-fold) compared to the control group (10.8 ± 4.4 vs. 5.4 ± 3.4; *p* = 0.015) (Table 2).

**Table 2:** The *SK3* expression in both the ganglionic and aganglionic colon of HSCR patient and control colon

SK3	ΔC_T_ ± SD	ΔΔC_T_ (95% CI)	Fold change	*p*-value
Ganglionic colon	9.9 ± 4.6	4.5 (0.1–8.9)	23	0.044*
Aganglionic colon	10.8 ± 4.4	5.4 (1.2–9.7)	43	0.015*
Control colon	5.4 ± 3.4			

*, *p* < 0.05 is considered statistically significant for the *SK3* expression difference between HSCR patient versus control colon

We followed-up all patients for a mean of 10.8 ± 16.0 months following pull-through. Three individuals (patient 4, 6, and 11) developed enterocolitis in 5, 18, and 2 months, respectively, after pull-through, while only one subject (patient 6) suffered post-operative soiling (Table 1). All symptoms resolved with rectal irrigations and administration of oral metronidazole.

## Discussion

We describe new data on Indonesian HSCR patients which reveal a similar frequency of short-segment aganglionosis as reported in the literature [17, 18]. Our study clearly demonstrates that the *SK3* expression was strongly reduced in the HSCR patient intestines, both in the ganglionic and aganglionic bowel, compared to the control intestines. These findings are consistent with previous observations [9, 19]. However, it should be noted that previous study [9] showed only half HSCR patients had reduced the *SK3* expression. These differences might be attributed to the differences in the genetic background of analyzed population between previous report [9] versus our study.

Persistent bowel symptoms might occur in about 10% of children following pull-through procedure. They range from constipation to severe episodes of abdominal distension, vomiting, and enterocolitis [20]. We eliminated the possibility of any residual aganglionosis in our patients with persistent bowel symptoms since we have performed intraoperative histopathological evaluations. There was no stricture identified in these patients after evaluation by a contrast enema.

It has been proposed that the down-regulated *SK3* in the HSCR colon results in unopposed cholinergic activity and a tonic hypercontraction, further causing a functional bowel obstruction in HSCR patients following pull-through [9]. Our study reveals a new evidence supporting this hypothesis by providing data from a genetically different population from previous reports [9, 19].

However, notably, the small sample size and limited power of the study, while a weakness of our report, implies that a significantly larger sample of patients needs to be involved to confirm our findings.

In this study, we involved the ARM patients as controls according to previous study [9]. It should be noted that most ARM patients suffer from a disturbance of the bowel motility [21]. Further study with more appropriate controls (e.g. trauma patients) is necessary to better clarify the impact of *SK3* expression in the persistent bowel symptoms after a definitive surgery.

Furthermore, the molecular pathogenesis of HSCR still needs to be elucidated. It might involve the compromised condition of genes responsible for gangliogenesis of the enteric nervous system (ENS) [5, 6, 12, 22], the neurotransmitters expressed by the neurons of ENS [11, 23] and their interactions. Our results clarify the role of *SK3* in the molecular pathogenesis of HSCR, particularly in Indonesia, a genetically distinct group within Asia [4–6, 12]. It is important to note that the down-regulation in *SK3* occurs in addition to other neurotransmitter signaling in HSCR. In addition, this study is the first report of *SK3* expression in a sample population of Asian ancestry. Moreover, our recent study shows that epistasis between *RET* and *NRG1* is important to ENS development [6]. It might be necessary to determine the *PDGFRA* expression level as a control and a marker for the PDGFRA+ cells that express *SK3* for better understanding the persistence bowel symptoms’ pathogenesis in HSCR patients following pull-through procedure. Unfortunately, we do not have any data on immunostaining of the resection segment for *SK3* due to limitation of resources in our institution. Furthermore, most patients (86%) in this cohort study carried the risk allele (T) for HSCR that are consistent with our previous findings [5, 6].

This study may contribute to extending the knowledge on mechanisms causing the persistence of bowel symptoms after an appropriately performed pull-through. The possibility of the continuing intestinal symptoms after an appropriately definitive surgery should be explained during surgery counseling to HSCR parents. Furthermore, the pediatric surgery involves more than conducting an operation but consists of total care of the patient and the family, of which proper diagnosis, prognosis and counseling are fundamental parts of the performance of procedure and the patient recovery process.

## Conclusion

Our study shows that the down-regulated *SK3* expression in ganglionic intestines might contribute to the persistent bowel symptoms following a properly performed pull-through surgery in Indonesian HSCR patients. Furthermore, this study is the first report of *SK3* expression in a sample population of Asian ancestry.

## Abbreviations

ARM: anorectal malformation
ENS: enteric nervous system
GAPDH: glyceraldehyde-3-phosphate dehydrogenase
HSCR: Hirschsprung disease
RT-PCR: real-time polymerase chain reaction
SK3: small-conductance calcium-activated potassium channel 3.

## References

[1] Chakravarti A, Lyonnet S, Scriver CR, Beaudet AL, Valle D, Sly WS, Childs B, Kinzler K, Vogelstein B (2001). Hirschsprung disease. The metabolic and molecular bases of inherited disease.

[2] Amiel J, Sproat-Emison E, Garcia-Barcelo M (2008). Hirschsprung disease, associated syndromes and genetics: a review. J Med Genet.

[3] Emison ES, Garcia-Barcelo M, Grice EA (2010). Differential contributions of rare and common, coding and noncoding ret mutations to multifactorial Hirschsprung disease liability. Am J Hum Genet.

[4] Abecasis GR, Altshuler D, 1000 Genomes Project Consortium (2010). A map of human genome variation from population-scale sequencing. Nature.

[5] Gunadi, Dwihantoro A, Iskandar K, Makhmudi A (2016). Rochadi. Accuracy of PCR-RFLP for RET rs2435357 genotyping as Hirschsprung risk. J Surg Res.

[6] Gunadi, Kapoor A, Ling AY (2014). Effects of RET and NRG1 polymorphisms in Indonesian patients with Hirschsprung disease. J Pediatr Surg.

[7] Zimmer J, Tomuschat C, Puri P (2016). Long-term results of transanal pull-through for Hirschsprung's disease: a meta-analysis. Pediatr Surg Int.

[8] Menezes M, Pini Prato A, Jasonni V (2008). Long-term clinical outcome in patients with total colonic aganglionosis: a 31-year review. J Pediatr Surg.

[9] Coyle D, O’Donnell AM, Puri P (2015). Altered distribution of small-conductance calcium-activated potassium channel SK3 in Hirschsprung’s disease. J Pediatr Surg.

[10] Kurahashi M, Zheng H, Dwyer L (2011). A functional role for the 'fibroblast-like cells' in gastrointestinal smooth muscles. J Physiol.

[11] Kurahashi M, Nakano Y, Hennig GW (2012). Platelet-derived growth factor receptor alpha-positive cells in the tunica muscularis of human colon. J Cell Mol Med.

[12] Gunadi, Makhmudi A, Agustriani N (2016). Rochadi. Effects of SEMA3 polymorphisms in Hirschsprung disease patients. Pediatr Surg Int.

[13] Setiadi JA, Dwihantoro A, Iskandar K, Heriyanto DS, Gunadi (2017). The utility of the hematoxylin and eosin staining in patients with suspected Hirschsprung disease. BMC Surg.

[14] Parahita IG, Makhmudi A, Gunadi. Comparison of Hirschsprung-associated enterocolitis following soave and Duhamel procedures. J Pediatr Surg. 2017; 10.1016/j.jpedsurg.2017.07.010. 10.1016/j.jpedsurg.2017.07.010 28755898

[15] Potier M, Joulin V, Roger S (2006). Identification of SK3 channel as a new mediator of breast cancer cell migration. Mol Cancer Ther.

[16] Livak KJ, Schmittgen TD (2001). Analysis of relative gene expression data using real-time quantitative PCR and the 2(−Delta Delta C(T)) method. Methods.

[17] Thakkar HS, Bassett C, Hsu A (2017). Functional outcomes in Hirschsprung disease: a single institution's 12-year experience. J Pediatr Surg.

[18] Langer JC, Coran AG (2012). Hirschsprung disease. Pediatric surgery.

[19] Piotrowska AP, Solari V, Puri P (2003). Distribution of Ca2+−activated K channels, SK2 and SK3, in the normal and Hirschsprung's disease bowel. J Pediatr Surg.

[20] Langer JC (2004). Persistent obstructive symptoms after surgery for Hirschsprung's disease: development of a diagnostic and therapeutic algorithm. J Pediatr Surg.

[21] Levitt MA, Peña A (2007). Anorectal malformations. Orphanet J Rare Dis.

[22] Alves MM, Sribudiani Y, Brouwer RW (2013). Contribution of rare and common variants determine complex diseases-Hirschsprung disease as a model. Dev Biol.

[23] O'Donnell AM, Coyle D, Puri P (2016). Decreased expression of NEDL2 in Hirschsprung's disease. J Pediatr Surg.

